# Draft Genome Sequences of Two Clinical Isolates of *Streptococcus mutans*

**DOI:** 10.1128/genomeA.00441-14

**Published:** 2014-06-12

**Authors:** Hui Zheng, Lihong Guo, Ning Du, Jiuxiang Lin, Lai Song, Guiming Liu, Feng Chen

**Affiliations:** a Department of Orthodontics, Peking University School and Hospital of Stomatology, Beijing, China; b Central of Laboratory, Peking University School and Hospital of Stomatology, Beijing, China; c CAS Key Laboratory of Genome Sciences and Information, Beijing Institute of Genomics, Chinese Academy of Sciences, Beijing, China

## Abstract

We report the draft genome sequences of PKUSS-HG01 and PKUSS-LG01, two clinical isolates of *Streptococcus mutans* from human dental plaque. The genomics information will facilitate the study of the mechanisms of pathogenicity and evolution of *S. mutans*.

## GENOME ANNOUNCEMENT

*Streptococcus mutans* is a Gram-positive organism that is the primary causative agent in the formation of dental caries in humans. *S. mutans* metabolizes a variety of carbohydrates, generating organic acid in dental plaque. The acidic environment results in mineral loss on the surface of teeth and eventually causes tooth decay (1). Furth more, it has been reported that *S. mutans* is implicated not only in infective endocarditis (2) but also in hemorrhagic stroke (3). To better understand the molecular basis of mechanisms of pathogenicity, degree of diversity, and genome rearrangement within the species, we sequenced the genomes of two strains from human dental plaque.

Whole-genome sequencing was performed using an Illumina Hi-Seq 2000 platform with an insert size of 500 bp, according to the manufacturer’s instructions. Paired sequences were generated, with total coverage of 315 and 310 for PKUSS-HG01 and PKUSS-LG01, respectively. For each genome, *de novo* assembly was performed using SOAPdenovo (4), and then the gaps within scaffolds were closed with GapFiller (5). Open reading frames (ORFs) were predicted with Glimmer 3.02 (6). Putative ribosomal binding sites were identified with RBSfinder (7). tRNAs and rRNAs were predicted using tRNAscan-SE (8) and RNAmmer (9), respectively. Functional annotations were performed by search against the NCBI nr, Swiss-Prot (10), Clusters of Orthologous Groups (COG) (11), KEGG (12), and InterProScan (13) databases.

We obtained 16 and 17 contigs larger than 500 bp for PKUSS-HG01 and PKUSS-LG01, respectively. The genomic sequence of PKUSS-HG01 comprises 1,951 coding sequences (CDSs), 3 rRNA loci, and 43 tRNAs, with a G+C content of 36.6%. In comparison, the genome sequence of PKUSS-LG01 contains 1,935 CDSs, 3 rRNA loci, and 39 tRNAs, with a G+C content of 36.6%.

Totals of 1,606 and 1,598 CDSs were assigned to COGs, and 1,064 and 1,057 CDSs can be annotated into 146 and 147 pathways by using KAAS (14) for PKUSS-HG01 and PKUSS-LG01, respectively. All-to-all BLASTP analysis with PKUSS-HG01 and PKUSS-LG01 protein sequences showed that they possesses 1,865 orthologous proteins. These genome sequences will enable further study of the mechanisms of pathogenicity and evolution and facilitate future drug development.

### Nucleotide sequence accession numbers.

This whole-genome shotgun project has been deposited at DDBJ/EMBL/GenBank under the accession number AXSW00000000 for strain PKUSS-HG01 and AXSX00000000 for strain PKUSS-LG01. The versions described in this paper are versions AXSW01000000 and AXSX01000000.
